# PsyNBIOsis: Investigating the Association between Maternal Gestational Diabetes, Mental Health, Diet and Childhood Obesity Risk: Protocol for a Prospective, Longitudinal, Observational Study

**DOI:** 10.3390/nu16010124

**Published:** 2023-12-29

**Authors:** Leah Gilbert, David Raubenheimer, Emily J. Hibbert, Ralph Nanan

**Affiliations:** 1 Nepean Clinical School, Faculty of Medicine and Health, University of Sydney, Penrith, NSW 2751, Australia; 2 Charles Perkins Centre, University of Sydney, Sydney, NSW 2003, Australia; 3 School of Life and Environmental Science, University of Sydney, Sydney, NSW 2003, Australia; 4 Nepean Hospital, Penrith, NSW 2747, Australia

**Keywords:** pregnancy, depression, anxiety, eating, breastfeeding, air displacement plethysmography system, human milk analysis

## Abstract

Background: Gestational diabetes mellitus (GDM) is associated with poorer maternal mental health (depression and anxiety). Maternal mental health and GDM are likely to influence diet, which in turn impacts the course of GDM. Maternal diet may also be directly or indirectly associated with changes in infant anthropometry. The aims of this study are to (1) examine the associations between maternal GDM, mental health and diet, and (2) evaluate the associations between these maternal factors, breastmilk composition and infant anthropometry. Methods: This prospective, observational, longitudinal cohort study compares a cohort of women with and without GDM. Maternal mental health and diet are assessed using validated questionnaires. Breastmilk composition is measured with the Human Milk Analyzer, and infant body composition is measured with air displacement plethysmography. Significance and Impact: Once data have been collected, PsyNBIOsis will provide evidence for the associations between maternal mental health, GDM status and diet, and their impact on breastmilk composition and early infant growth. The results may inform the Developmental Origins of Health and Disease framework and provide data on which to build cost-effective interventions to prevent both the development of mental health issues in mothers and adverse growth patterns in infants.

## 1. Introduction

Gestational diabetes mellitus (GDM) occurs in 10.13% of pregnancies worldwide [1]. Depression and anxiety are the most common mental health disorders in pregnancy and in the postpartum period, with prevalence ranging between 7 and 30% for depression and 7 and 20% for anxiety [2]. Poor maternal mental health and GDM coincide, both throughout pregnancy and extending to the postpartum period. Symptoms of depression or anxiety in early pregnancy are associated with a higher risk of being diagnosed with GDM; conversely, GDM diagnosis is associated with a higher risk of developing symptoms of depression and/or anxiety in pregnancy and the postpartum period [3,4]. 

Maternal GDM, anxiety and depression are associated with adverse changes in maternal diet, such as changes in macronutrient intake, although study findings are inconsistent [5,6,7,8]. These changes in maternal diet could be important factors contributing to adverse growth in infants. Firstly, maternal diet may impact the offspring’s growth in utero. Changes in macronutrients in pregnancy such as energy restriction (reductions in the total amount of food intake) are associated with a risk of fetal growth restriction [9]. Secondly, exposure to certain macronutrients in utero has the potential to shape the food preferences of the offspring, such as searching reward sensations through highly palatable foods, which are associated with an increased risk of obesity [10,11]. However, the relationship between maternal diet and fetal growth is complex and many aspects remain unclear, especially regarding the role of protein [12,13,14]. Thirdly, maternal diet also shapes the postpartum food environment of the offspring as it is directly associated with the macronutrients present in breastmilk, which in turn contribute to the child’s growth [15,16]. 

Obesity rates in children are growing worldwide, and infants born to pregnancies where mothers are diagnosed with GDM are at an even greater risk of obesity than pregnancies without GDM [17,18,19,20]. Therefore, it is important to understand the association between maternal mental health, GDM and maternal diet since growth patterns in utero and early infancy are determinants of obesity and associated conditions in later life [21]. So far, studies linking maternal mental health and nutrition in pregnancy show that women with depression and anxiety have a higher intake of overall energy than women without mental health symptoms [5,6,7]. Studies on maternal diet, especially studies investigating macronutrient intake in women with GDM, show conflicting findings. One study demonstrated changes in maternal diet in the weeks following a GDM diagnosis, with a lower intake of energy (kcal), a lower proportion of carbohydrates and a higher proportion of protein intake compared to women without GDM [22]. In contrast, another found that, after a GDM diagnosis, women with GDM have a lower proportion of carbohydrates and lower total energy intake but no changes in the proportion of protein intake in both longitudinal and cross-sectional comparisons [23]. These inconsistencies regarding the specific changes in the proportion of protein intake after a GDM diagnosis need to be investigated further, as changes in protein intake may potentially be associated with mental health issues and contribute to harmful effects on fetal growth [9,24]. Examining associations between maternal GDM, mental health and early infant growth constitutes the primary aim of our study, as maternal mental health and diet constitute the most promising pillars on which to intervene to improve both maternal and infant mental and metabolic health [25].

As mentioned above, maternal diet may influence the macronutrients present in breastmilk and, therefore, the growth of the infant. Regarding the impact of GDM, only one study shows changes in breastmilk composition in the context of maternal GDM [15]. No studies have investigated the association between maternal mental health issues and changes in breastmilk composition. Evaluating the association between maternal factors (mental health, GDM and diet), breastmilk composition and infant anthropometry will constitute the second aim of our study.

Finally, other behavioral factors such as maternal and infant eating behaviors, maternal breastfeeding behaviors and maternal and infant sleeping behaviors may be associated with maternal GDM, mental health status, diet, breastmilk composition and infant growth, and thus need to be explored further [5,26,27,28,29,30,31,32,33,34,35,36,37,38,39,40,41,42,43,44,45,46,47]. 

In this brief report, our objective is to publish a protocol designed to achieve the following aims:

1. Examine the associations between maternal mental health, GDM status and maternal overall diet, focusing on specific changes in macronutrient proportions with a special emphasis on the percent of energy from protein;
2. Evaluate the association between maternal factors (mental health, GDM status, diet), breastmilk composition and infant anthropometry;
3. Ascertain how behavioral aspects in the mother and the infant, such as eating, breastfeeding and sleeping behaviors, may be associated with maternal mental health, GDM, diet, breastmilk composition and infant anthropometry.

This PsyNBIOsis study protocol firstly highlights the important gaps that remain in the literature regarding the consideration of maternal mental health issues and GDM and their association with maternal diet, breastmilk composition and, thus, their potential important transgenerational impact on infant obesity risk. This protocol also brings forward other behavioral and complex factors, such as maternal and infant eating and sleeping behaviors, and their complex interaction in the perinatal period. To our knowledge, this is the first study that will integrate psychological, nutritional and behavioral factors to understand infant obesity risk. Our protocol employs state-of-the-art methods, including gold standard techniques to assess breastmilk composition and infant body composition, and nutritional geometry to interpret dietary intakes and their relationships with outcomes [48]. Our goal in sharing this protocol is to enable it to be used, modified, or discussed by other researchers wanting to analyze the complex yet understudied impact of mental health and behaviors on infant obesity risk.

## 2. Materials and Methods

### 2.1. Trial Design

This study is a prospective, longitudinal, observational cohort study, approved by the Nepean Blue Mountains Local Health District (NBMHD) Human Research Ethics Committee (HREC) 2022/ETH00326.

### 2.2. Study Setting, Recruitment, Consent, Eligibility and Group Allocation

The PsyNBIOsis study is being conducted at Nepean Hospital, a tertiary hospital in Western Sydney, New South Wales, Australia. Nepean Hospital is the largest hospital of six in the Nepean Blue Mountains Local Health District (NBMLHD). All women from this geographically diverse LHD who have high-risk pregnancies are seen at the Nepean Hospital antenatal clinic. The LHD encompasses rural and outer-metropolitan areas and has pockets of extreme socio-economic disadvantage.

**Figure 1:**
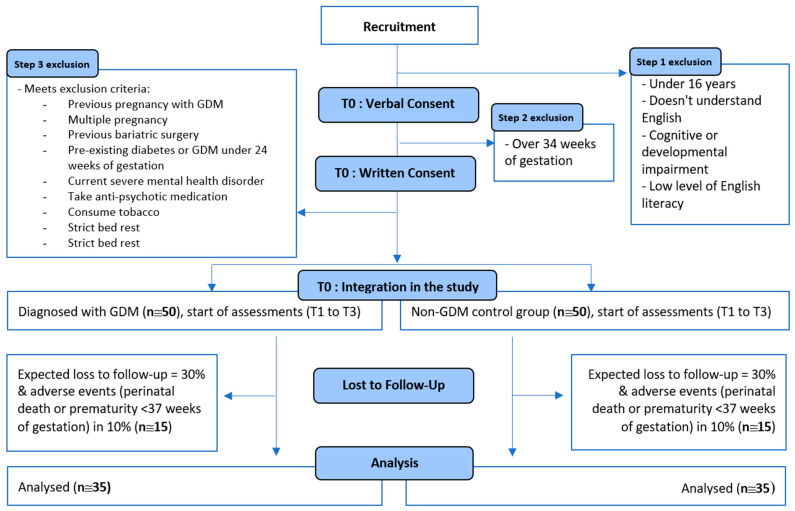
Flow chart depicting the design, exclusion criteria and loss to follow-up planned for the PsyNBIOsis study.

Women are approached during routine antenatal visits, and the study is briefly explained. Women are eligible if they are 16 years of age or older, understand English, have no cognitive impairment, have sufficient health literacy to understand the questionnaires and thus can verbally consent to the study. They are asked to provide written consent via REDCap (Research Electronic Data Capture), a secure, web-based data collection tool which allows data to be stored in the format of a survey or database [49,50]. Next, these women complete an online eligibility questionnaire to ensure that they meet the inclusion and no exclusion criteria. Women who consent, are eligible and are diagnosed with GDM are allocated to the GDM group, and women who have not been diagnosed with GDM are allocated to the control group. The exclusion criteria are detailed in Figure 1.

### 2.3. Sample Size

The sample size is determined based on preliminary calculations showing a statistically significant difference in the percent of total energy intake from protein between the GDM and control group. The third timepoint (8 to 12 weeks postpartum) of this study will be used as the basis for the analysis of this difference. This is because this study aims to answer the inconsistencies that exist in the literature regarding percent protein intake in mothers after a GDM diagnosis, i.e., the perinatal period [22,23]. The first timepoint of PsyNBIOsis would have only enabled us to measure differences in diet during pregnancy, and thus does not form the basis for our power calculation. The required sample size is around 50 women in each study group at inclusion to have a statistically significant difference (*p* = 0.05) in the percentage of total energy intake from protein between the GDM and the control group with a power of 80% at the third time point. Based on previous research, this sample size is also sufficient to observe statistically significant differences in depression and anxiety between the GDM and control groups [3,4], and to observe associations between maternal diet and mental health (depression and anxiety) [6]. A 30% loss to follow-up is expected based on a similar study collecting breastmilk in women with and without GDM [51]. Adverse events (e.g., prematurity) leading to study withdrawal and women not breastfeeding at our last time point are expected in 10% of the population [52,53].

### 2.4. Data Collection and Study Visits

Once consented and found to meet all eligibility criteria (study inclusion, T0), maternal mental health; diet; eating and sleeping behaviors; and sociodemographic information (such as educational level and parity) are assessed through REDCap online questionnaires (T1). In addition, the women’s GDM status is retrieved from their electronic medical record, and the women are allocated to the corresponding groups.

At 0–1 week postpartum (T2), women are asked to complete further online questionnaires via REDCap assessing maternal mental health; eating, breastfeeding and sleeping behaviors; obstetric and neonatal information; and infant eating and sleeping behaviors. Infant anthropometry is measured, retrieved from electronic medical records or self-reported by the mothers. 

At 8–12 weeks postpartum (T3), the measures from T1 and T2 are repeated. Infant anthropometry is measured or self-reported by the mothers, and the postpartum maternal oral glucose tolerance test (oGTT) results are retrieved for women from the GDM group. For women who are breastfeeding, breastmilk composition is assessed through human milk analysis. 

Recruitment for this study started on 1 November 2022.

### 2.5. Measures

The surveys being used in this study are all well validated in the perinatal period [35,36,54,55,56,57,58,59,60,61,62,63,64] and, for most of them, also in Australian populations [65,66,67,68,69,70,71,72,73,74]. All questionnaires are filled out by the mothers on REDCap [49,50].

#### 2.5.1. Maternal Mental Health

**Figure 2:**
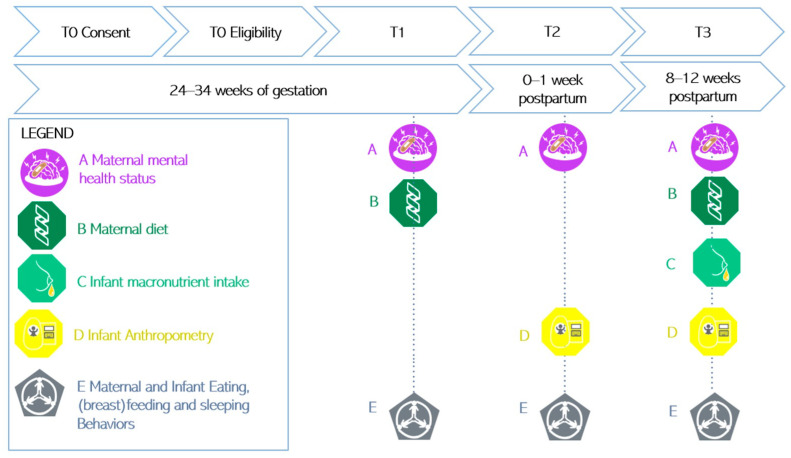
Illustration of the measures at each time point.

The Edinburgh Postnatal Depression Scale (EPDS) is used to measure the presence of symptoms of depression in the past 7 days through 10 items scored from 0 to 3 [75]. The Perinatal Anxiety and Stress Scale (PASS) is used to measure the presence of symptoms of anxiety in the past month, with four subscales assessing acute anxiety and adjustment, general worry and specific fears, perfectionism, control, trauma and social anxiety, with 31 items scored from 0 to 3 [56,76]; see Figure 2, letter A.

#### 2.5.2. Maternal Diet

Maternal dietary intake is measured with the Australian Eating Survey (AES), a 120-item web-based food frequency questionnaire that measures macro- and micronutrient intake, such as protein, and contains questions about the frequency of consumption of takeaway food and vitamin supplements over the past 6 months [77,78]; see Figure 2, letter B.

#### 2.5.3. Breastmilk Composition

Breastmilk composition (fat, lactose, protein, true protein, oligosaccharides and energy) is assessed with the Miris Human Milk Analyzer (HMA). One sample of 3 to 5 mL of both fore and hind breastmilk are expressed and collected in the morning [65]. Breastmilk is stored at −80 °C before it is defrosted in a water bath and maintained at 40 °C for sonication and analysis. As recommended by Leghi et al., the breast from which milk was collected, the timing of collection, the timing of the last feed prior to extraction, as well as the method used (breast pump or manual extraction) is recorded for both the fore and hind milk [79]. For infants who are formula-fed, information is retrieved on the formula brand the participant is using; see Figure 2, letter C.

#### 2.5.4. Infant Anthropometric Outcomes

The infant’s length is measured to the nearest 0.1 cm with a measuring board at T2 and T3 [80]. Infant weight, fat and fat-free mass are measured with the Pea Pod instrument. The Pea Pod is a non-invasive air displacement plethysmography system, using whole-body densitometry to determine body composition (fat and fat-free mass) in infants. The accuracy and precision of the Pea Pod has been demonstrated in previous research, in various infant populations [81,82], and is considered a non-invasive gold standard technique for the measurement of infant body composition. The infant’s weight and length percentiles and z-scores are determined with standard criteria [83,84]. Additionally, mothers are asked for the last measured infant weight and length on the REDCap questionnaires [50] so that, if they miss the appointment, we still have self-reported data; see Figure 2, letter D.

#### 2.5.5. Maternal and Infant Eating Behavior

Maternal eating behavior is assessed with the Intuitive Eating Scale (IES-2) at all timepoints. The IES-2 is a 23-item questionnaire that assesses an individual’s eating behaviors with four subscales: unconditional permission to eat, eating for physical rather than emotional reasons, reliance on hunger/satiety cues and body–food choice congruence. Infant eating behavior is assessed with the Baby Eating Behavior Questionnaire (BEBQ) at T2 and T3. This is a self-report questionnaire that assesses the infant’s eating behaviors with 18 items and four subscales: food and satiety responsiveness, enjoyment of food and slowness in eating [60]. 

#### 2.5.6. Maternal Breastfeeding Behavior

Maternal breastfeeding behavior is assessed with the Infant Feeding Style Questionnaire (IFSQ) and the Food to Soothe Questionnaire (FTSQ) at T2 and T3. The IFSQ consists of three subscales containing 83 items. We are using only two of these subscales (54 items), those measuring maternal beliefs and behaviors in infants (e.g., laissez-faire, restrictive, pressuring, responsive and indulgent feeding), as the last subscale measures behaviors relating to the intake of solid foods, which is not relevant to this study [85].

#### 2.5.7. Maternal and Infant Sleeping Behavior

Maternal sleeping behavior is assessed with the Pittsburgh Sleep Quality Index (PSQI) at all time points. The PSQI is used to measure sleep disturbance and usual sleep behavior in the past month, with 19 items scored on a 0–3-point Likert scale [86]. Infant sleeping behavior is assessed with the Brief Infant Sleep Questionnaire—Revised (BISQ-R) at T2 and T3. The BISQ-R is an age-based norm-referenced scoring system to measure infant sleep behavior in the past 2 weeks through 19 items and 3 subscales: infant sleep quality, parent perception of infant sleep and parent behaviors that promote healthy and independent sleep [62]. 

#### 2.5.8. Covariates

GDM information and oGTT results (T2 and T3): Information for participants with GDM regarding GDM management, i.e., whether their glucose was controlled through diet, insulin and/or metformin and post-partum oGTT glucose values at 6–12 weeks, are collected from electronic medical records or from the participants themselves.

Maternal anthropometry (at all time points): Maternal weight is measured to the nearest 0.01 kg and height to the nearest 0.1 cm or self-reported via REDCap [50,56]. Maternal BMI is determined by the formula kg/m^2^ and BMI categories are determined according to ethnicity [87,88,89]. Additionally, maternal pre-pregnancy weight is retrieved from electronic medical records. Maternal gestational weight gain up to study inclusion, as well as whether women follow the Institute of Medicine recommendations for gestational weight gain, will be determined based on standard criteria [90].

Socio-demographic information (T1): Participants are asked via the online questionnaires about their education, job, family history of diabetes, partner status and whether they feel that they have emotional and instrumental social support. Information on gravida, parity and domestic violence is also retrieved from the participant’s medical record.

Obstetric and neonatal outcomes (T2): Information is collected from the participants on the date of birth, birth order and mode of delivery of the infants and birth weight of previous infants—if there are any.

Food Insecurity (T3): Two items are assessed at the end of the study to evaluate food insecurity over the whole study period [64]. 

### 2.6. Planned Analysis

For our first aim, examining the association between maternal status (depression and/or anxiety and GDM), maternal overall diet and specific changes in macronutrient proportions, we will first perform structural equation modelling (SEM) [91]. SEM will enable us to determine associations between our variables of interest [91]. Secondly, we will evaluate differences in the percent of energy from protein in maternal diet by using the Geometric Framework for Nutrition (GFN) [92]. 

To investigate our second aim, we will evaluate the association between maternal factors (maternal depression, anxiety, GDM status and diet), breastmilk composition and infant anthropometry through SEM. Secondly, we will conduct a moderation analysis to establish whether stratified analyses need to be conducted according to infant sex. Thirdly, the GFN will allow a representation of the topologies of diets and their impact on infant anthropometry [48].

Finally, for our third aim, we will ascertain if maternal mental health, GDM, diet, breastmilk composition and infant anthropometry are associated with behaviors such as maternal and infant eating, breastfeeding and sleeping behavior. 

Variables will be transformed if residuals are not normally distributed, and analyses will be undertaken with R [93]. In all analyses, we will control for important covariates where appropriate.

## 3. Anticipated Results

As illustrated in our graphical abstract, we hypothesize the following:

1. There will be associations between maternal depression, anxiety and GDM status and diet.
2. Maternal depression, anxiety, GDM status and diet will be associated with breastmilk composition and infant anthropometric outcomes.
3. Behaviors such as eating, breastfeeding and sleeping will be associated with maternal mental health, GDM status, diet, breastmilk composition and infant anthropometric outcomes.

## 4. Discussion

This prospective, observational, longitudinal cohort study will provide novel information about the association between GDM, mental health and macronutrient proportions and absolute intake during pregnancy and the early postpartum period. Secondly, the study will evaluate the association between maternal factors (maternal depression, anxiety, GDM status and diet), breastmilk composition and infant anthropometry. The PsyNBIOsis study will determine which modifiable risk factors are associated with a higher risk of infant obesity in populations with and without depression, anxiety and GDM. This will provide baseline data to inform the development of future comprehensive, tailored psychological and nutritional interventions to reduce the risk of maternal mental health issues and obesity in infants, and to reduce their related health and economic costs [94,95,96]. Therefore, PsyNBIOsis is likely to generate findings that will inform and influence the practice of obesity prevention across a broad range of healthcare professions including endocrinology, psychology, nutrition, neonatology and pediatrics.

### Limitations and Ethical Considerations

The main limitation of this study is the short period of follow-up currently planned, extending to only 12 weeks postpartum. Indeed, even if early infant growth is an important predictor of infant obesity; it is ideal to measure growth patterns in the first 15 months [21]. However, women are asked, during the consent process, whether they agree to be contacted in the future in relation to this study. This provides the option of a follow-up study to measure longer-term outcomes. Secondly, elements such as mental health, dietary intake and behaviors are measured through self-reported online questionnaires and would benefit from being measured through gold standard techniques such as interviews with clinical psychologists and dieticians [97]. Finally, a mother’s physical activity may influence both her mental health [98] and the infant’s growth [99]. However, physical activity is not assessed in the PsyNBIOsis study and thus may be considered as a limitation.

## Data Availability

The datasets generated by this study are available, upon request, from the first author, until they are placed on a public repository at study completion.

## References

[1] Gyasi-Antwi P., Walker L., Moody C., Moody C., Okyere S., Salt K., Anang L., Eduful E., Laryea D., Ottie-Boakye D. (2020). Global Prevalence of Gestational Diabetes Mellitus: A Systematic Review and Meta-Analysis. New Am. J. Med..

[2] Biaggi A., Conroy S., Pawlby S., Pariante C.M. (2016). Identifying the women at risk of antenatal anxiety and depression: A systematic review. J. Affect. Disord..

[3] Wilson C.A., Newham J., Rankin J., Ismail K., Simonoff E., Reynolds R.M., Stoll N., Howard L.-M. (2020). Is there an increased risk of perinatal mental disorder in women with gestational diabetes? A systematic review and meta-analysis. Diabet. Med..

[4] OuYang H., Chen B., Abdulrahman A.-M., Li L., Wu N. (2021). Associations between Gestational Diabetes and Anxiety or Depression: A Systematic Review. J. Diabetes Res..

[5] Hurley K.M., Caulfield L.E., Sacco L.M., Costigan K.A., Dipietro J.A. (2005). Psychosocial Influences in Dietary Patterns during Pregnancy. J. Am. Diet. Assoc..

[6] Fowles E.R., Murphey C., Ruiz R.J. (2011). Exploring Relationships among Psychosocial Status, Dietary Quality, and Measures of Placental Development during the First Trimester in Low-Income Women. Biol. Res. Nurs..

[7] Singh A., Trumpff C., Genkinger J., Davis A., Spann M., Werner E., Monk C. (2017). Micronutrient Dietary Intake in Latina Pregnant Adolescents and Its Association with Level of Depression, Stress, and Social Support. Nutrients.

[8] Oh J., Yun K., Chae J.-H., Kim T.-S. (2020). Association between Macronutrients Intake and Depression in the United States and South Korea. Front. Psychiatry.

[9] Kramer M.S., Kakuma R., Kramer M.S. (2003). Energy and protein intake in pregnancy. Cochrane Database of Systematic Reviews.

[10] Muhlhausler B.S., Ong Z.Y. (2011). The Fetal Origins of Obesity: Early Origins of Altered Food Intake. Endocr. Metab. Immune Disord. Drug Targets.

[11] Bischoff A.R., DalleMolle R., Silveira P.P. (2017). Fetal Programming of Food Preferences and Feeding Behavior. Diet, Nutrition, and Fetal Programming.

[12] Kind K.L., Moore V.M., Davies M.J. (2006). Diet around conception and during pregnancy—Effects on fetal and neonatal outcomes. Reprod. Biomed. Online.

[13] Zeisel S.H. (2009). Is maternal diet supplementation beneficial? Optimal development of infant depends on mother’s diet. Am. J. Clin. Nutr..

[14] Mousa A., Naqash A., Lim S. (2019). Macronutrient and Micronutrient Intake during Pregnancy: An Overview of Recent Evidence. Nutrients.

[15] Komatsu Y., Wada Y., Tabata F., Kawakami S., Takeda Y., Nakamura K., Ayabe T., Nakamura K., Kimura T., Tamakoshi A. (2023). Associations between Maternal Diet, Human Milk Macronutrients, and Breast-Fed Infant Growth during the First Month of Life in the SMILE Iwamizawa in Japan. Nutrients.

[16] De Paula M.V.Q., Grant M., Lanigan J., Singhal A. (2023). Does human milk composition predict later risk of obesity? A systematic review. BMC Nutr..

[17] Nehring I., Chmitorz A., Reulen H., von Kries R., Ensenauer R. (2013). Gestational diabetes predicts the risk of childhood overweight and abdominal circumference independent of maternal obesity. Diabet. Med..

[18] Mehta S.H., Kruger M., Sokol R.J. (2012). Is maternal diabetes a risk factor for childhood obesity?. J. Matern. Fetal Neonatal Med..

[19] Zhao P., Liu E., Qiao Y., Katzmarzyk P.T., Chaput J.-P., Fogelholm M., Johnson W., Kuriyan R., Kurpad A., Lambert E.V. (2016). Maternal gestational diabetes and childhood obesity at age 9–11: Results of a multinational study. Diabetologia.

[20] Wang Y., Lobstein T. (2006). Worldwide trends in childhood overweight and obesity. Int. J. Pediatr. Obes..

[21] Gittner L.S., Ludington-Hoe S.M., Haller H.S. (2013). Utilising infant growth to predict obesity status at 5 years. J. Paediatr. Child. Health.

[22] Atakora L., Poston L., Hayes L., Flynn A.C., White S.L. (2020). Influence of GDM Diagnosis and Treatment on Weight Gain, Dietary Intake and Physical Activity in Pregnant Women with Obesity: Secondary Analysis of the UPBEAT Study. Nutrients.

[23] Hinkle S.N., Li M., Grewal J., Yisahak S.F., Grobman W.A., Newman R.B., Wing D.A., Grantz K.L., Zhang C. (2021). Changes in Diet and Exercise in Pregnant Women after Diagnosis with Gestational Diabetes: Findings from a Longitudinal Prospective Cohort Study. J. Acad. Nutr. Diet..

[24] Chen L.-W., Aris I.M., Bernard J.Y., Tint M.-T., Colega M., Gluckman P.D., Tan K.H., Shek L.P.-C., Chong Y.-S., Yap F. (2017). Associations of maternal macronutrient intake during pregnancy with infant BMI peak characteristics and childhood BMI. Am. J. Clin. Nutr..

[25] Holmes E.A., Ghaderi A., Harmer C.J., Ramchandani P.G., Cuijpers P., Morrison A.P., Roiser J.P., Bockting C.L.H., O’Connor R.C., Shafran R. (2018). The Lancet Psychiatry Commission on psychological treatments research in tomorrow’s science. Lancet Psychiatry.

[26] Quansah D.Y., Gross J., Gilbert L., Helbling C., Horsch A., Puder J.J. (2019). Intuitive eating is associated with weight and glucose control during pregnancy and in the early postpartum period in women with gestational diabetes mellitus (GDM): A clinical cohort study. Eat. Behav..

[27] Khalsa A.S., Woo J.G., Kharofa R.Y., Geraghty S.R., DeWitt T.G., Copeland K.A. (2019). Parental intuitive eating behaviors and their association with infant feeding styles among low-income families. Eat. Behav..

[28] Van Dyke N., Drinkwater E.J. (2014). Review Article Relationships between intuitive eating and health indicators: Literature review. Public Health Nutr..

[29] Craig L., Sims R., Glasziou P., Thomas R. (2020). Women’s experiences of a diagnosis of gestational diabetes mellitus: A systematic review. BMC Pregnancy Childbirth.

[30] Catalano P.M., Kirwan J.P., Mouzon S.H.-D., King J. (2003). Gestational Diabetes and Insulin Resistance: Role in Short- and Long-Term Implications for Mother and Fetus. J. Nutr..

[31] Farrow C.V., Blissett J.M. (2005). Is Maternal Psychopathology Related to Obesigenic Feeding Practices at 1 Year?. Obes. Res..

[32] Manerkar K., Harding J., Conlon C., McKinlay C. (2020). Maternal gestational diabetes and infant feeding, nutrition and growth: A systematic review and meta-analysis. Br. J. Nutr..

[33] Suwaydi M.A., Wlodek M.E., Lai C.T., Prosser S.A., Geddes D.T., Perrella S.L. (2022). Delayed secretory activation and low milk production in women with gestational diabetes: A case series. BMC Pregnancy Childbirth.

[34] Jansen E., Naymik M., Thapaliya G., Huentelman M., Beauchemin J., D’Sa V., Lewis C.R., Deoni S., Carnell S., RESONANCE Consortium (2023). Parent-reported child appetite moderates relationships between child genetic obesity risk and parental feeding practices. Front. Nutr..

[35] Stifter C.A., Anzman-Frasca S., Birch L.L., Voegtline K. (2011). Parent use of food to soothe infant/toddler distress and child weight status. An exploratory study. Appetite.

[36] Thompson A.L., Bentley M.E. (2013). The critical period of infant feeding for the development of early disparities in obesity. Soc. Sci. Med..

[37] Patel N., Dalrymple K.V., Briley A.L. (2018). Pasupathy, D.; Seed, P.T.; Flynn, A.C.; Poston, L. Mode of infant feeding, eating behaviour and anthropometry in infants at 6-months of age born to obese women—A secondary analysis of the UPBEAT trial. BMC Pregnancy Childbirth.

[38] Olga L., van Diepen J.A., Gross G., Dunger D.B., Ong K.K. (2022). Early weight gain influences duration of breast feeding: Prospective cohort study. Arch. Dis. Child..

[39] Cheshmeh S., Nachvak S.M., Hojati N., Elahi N., Heidarzadeh-Esfahani N., Saber A. (2022). The effects of breastfeeding and formula feeding on the metabolic factors and the expression level of obesity and diabetes-predisposing genes in healthy infants. Physiol. Rep..

[40] Reutrakul S., Chen H., Chirakalwasan N., Charoensri S., Wanitcharoenkul E., Amnakkittikul S., Saetung S., Layden B.T., Chlipala G.E. (2021). Metabolomic profile associated with obstructive sleep apnoea severity in obese pregnant women with gestational diabetes mellitus: A pilot study. J. Sleep. Res..

[41] Ma S., Yin X., Tao R., Jiang X., Xie J., Li P., Zhu D., Zhu P. (2022). Association of maternal prenatal depression and anxiety with toddler sleep: The China-Anhui Birth Cohort study. Arch. Womens Ment. Health.

[42] Okun M.L. (2015). Sleep and postpartum depression. Curr. Opin. Psychiatry.

[43] Balserak B.I. (2015). Sleep disordered breathing in pregnancy. Breathe.

[44] Gilbert L., Sandoz V., Quansah D.Y., Puder J.J., Horsch A. (2022). Prospective Associations between Maternal Depression and Infant Sleep in Women with Gestational Diabetes Mellitus. Front. Psychol..

[45] Liu H., Xia W., Xiong X., Li J.-X., Li Y., Xu S.-Q., Li Y.-Y. (2021). Associations between Maternal Sleep Quality Throughout Pregnancy and Newborn Birth Weight. Behav. Sleep. Med..

[46] Miller M.A., Kruisbrink M., Wallace J., Ji C., Cappuccio F.P. (2018). Sleep duration and incidence of obesity in infants, children, and adolescents: A systematic review and meta-analysis of prospective studies. Sleep..

[47] Yisahak S.F., Boone K.M., Rausch J., Keim S.A. (2023). The timing and quality of sleep was associated with dietary quality and anthropometry in toddlers born preterm. Acta Paediatr..

[48] Simpson S.J., Le Couteur D.G., James D.E., George J., Gunton J.E., Solon-Biet S.M., Raubenheimer D. (2017). The Geometric Framework for Nutrition as a tool in precision medicine. Nutr. Healthy Aging.

[49] Harris P.A., Taylor R., Thielke R., Payne J., Gonzalez N., Conde J.G. (2009). Research electronic data capture (REDCap)—A metadata-driven methodology and workflow process for providing translational research informatics support. J. Biomed. Inform..

[50] Harris P.A., Taylor R., Minor B.L., Elliott V., Fernandez M., O’Neal L., McLeod L., Delacqua G., Delacqua F., Kirby J. (2019). The REDCap consortium: Building an international community of software platform partners. J. Biomed. Inform..

[51] Klein K., Bancher-Todesca D., Graf T., Garo F., Roth E., Kautzky-Willer A., Worda C. (2013). Concentration of Free Amino Acids in Human Milk of Women with Gestational Diabetes Mellitus and Healthy Women. Breastfeed. Med..

[52] Wan C.S., Abell S., Aroni R., Nankervis A., Boyle J., Teede H. (2019). Ethnic differences in prevalence, risk factors, and perinatal outcomes of gestational diabetes mellitus: A comparison between immigrant ethnic Chinese women and Australian-born Caucasian women in Australia. J. Diabetes.

[53] Internet Australian Bureau of Statistics Breastfeeding. https://www.abs.gov.au/statistics/health/health-conditions-and-risks/breastfeeding/latest-release.

[54] Bunevicius A., Kusminskas L., Pop V.J., Pedersen C.A., Bunevicius R. (2009). Screening for antenatal depression with the Edinburgh Depression Scale. J. Psychosom. Obstet. Gynecol..

[55] Navarro P., Ascaso C., Garcia-Esteve L., Aguado J., Torres A., Martín-Santos R. (2007). Postnatal psychiatric morbidity: A validation study of the GHQ-12 and the EPDS as screening tools. Gen. Hosp. Psychiatry.

[56] Somerville S., Ascaso C., Garcia-Esteve L., Aguado J., Torres A., Martín-Santos R. (2014). The Perinatal Anxiety Screening Scale: Development and preliminary validation. Arch. Womens Ment. Health.

[57] Ashman A.M., Collins C.E., Hure A.J., Jensen M., Oldmeadow C. (2016). Maternal diet during early childhood, but not pregnancy, predicts diet quality and fruit and vegetable acceptance in offspring. Matern. Child Nutr.

[58] Daundasekara S.S., Beasley A.D., O’Connor D.P., Sampson M., Hernandez D., Ledoux T. (2017). Validation of the intuitive Eating Scale for pregnant women. Appetite.

[59] Savard C., Yan E., Plante A.-S., Bégin C., Robitaille J., Michaud A., Lemieux S., Provencher V., Morisset A.-S. (2020). Positive attitudes toward weight gain in late pregnancy are associated with healthy eating behaviours. Eat. Weight. Disord. Stud. Anorex. Bulim. Obes..

[60] Llewellyn C.H., van Jaarsveld C.H.M., Johnson L., Carnell S., Wardle J. (2011). Development and factor structure of the Baby Eating Behaviour Questionnaire in the Gemini birth cohort. Appetite.

[61] Plows J.F., Berger P.K., Jones Roshonda B., Yonemitsu C., Ryoo J.H., Alderete T.L., Bode L., Goran M.I. (2020). Associations between human milk oligosaccharides (HMOs) and eating behaviour in Hispanic infants at 1 and 6 months of age. Pediatr. Obes..

[62] Mindell J.A., Gould R.A., Tikotzy L., Leichman E.S., Walters R.M. (2019). Norm-referenced scoring system for the Brief Infant Sleep Questionnaire—Revised (BISQ-R). Sleep Med..

[63] Meltzer L.J., Paisley C. (2023). Beyond Polysomnography. Sleep Med. Clin..

[64] Gundersen C., Engelhard E.E., Crumbaugh A.S., Seligman H.K. (2017). Brief assessment of food insecurity accurately identifies high-risk US adults. Public Health Nutr..

[65] Menjo A., Mizuno K., Murase M., Nishida Y., Taki M., Itabashi K., Shimono T., Namba K. (2009). Bedside analysis of human milk for adjustable nutrition strategy. Acta Paediatr..

[66] Borràs-Novell C., Herranz Barbero A., Aldecoa-Bilbao V., Feixas Orellana G., Balcells Esponera C., Sánchez Ortiz E., García-Algar O., Iglesias Platas I. (2020). Infrared analyzers for the measurement of breastmilk macronutrient content in the clinical setting. Expert Rev. Mol. Diagn..

[67] Boyce P., Stubbs J., Todd A. (1993). The Edinburgh Postnatal Depression Scale: Validation for an Australian Sample. Aust. N. Z. J. Psychiatry.

[68] Strodl E., Markey C., Aimé A., Rodgers R.F., Dion J., Coco G.L., Gullo S., McCabe M., Mellor D., Granero-Gallegos A. (2020). A cross-country examination of emotional eating, restrained eating and intuitive eating: Measurement Invariance across eight countries. Body Image.

[69] Mallan K.M., Daniels L.A., de Jersey S.J. (2014). Confirmatory factor analysis of the Baby Eating Behaviour Questionnaire and associations with infant weight, gender and feeding mode in an Australian sample. Appetite.

[70] Backhaus J., Junghanns K., Broocks A., Riemann D., Hohagen F. (2002). Test–retest reliability and validity of the Pittsburgh Sleep Quality Index in primary insomnia. J. Psychosom. Res..

[71] Skouteris H., Wertheim E.H., Germano C., Paxton S.J., Milgrom J. (2009). Assessing Sleep during Pregnancy. Women’s Health Issues.

[72] Sakalidis V.S., Rea A., Perrella S.L., McEachran J., Collis G., Miraudo J., Prosser S.A., Gibson L.Y., Silva D., Geddes D.T. (2021). Wellbeing of Breastfeeding Women in Australia and New Zealand during the COVID-19 Pandemic: A Cross-Sectional Study. Nutrients.

[73] Quin N., Tikotzky L., Stafford L., Fisher J., Bei B. (2022). Preventing postpartum insomnia by targeting maternal versus infant sleep: A protocol for a randomized controlled trial (the Study for Mother-Infant Sleep ‘SMILE’). Sleep Adv..

[74] Zinga J., McKay F.H., Lindberg R., van der Pligt P. (2022). Experiences of Food-Insecure Pregnant Women and Factors Influencing Their Food Choices. Matern. Child Health J..

[75] Cox J.L., Holden J.M., Sagovsky R. (1987). Detection of Postnatal Depression. Br. J. Psychiatry.

[76] Somerville S., Byrne S.L., Dedman K., Hagan R., Coo S., Oxnam E., Doherty D., Cunningham N., Page A.C. (2015). Detecting the severity of perinatal anxiety with the Perinatal Anxiety Screening Scale (PASS). J. Affect. Disord..

[77] Collins C.E., Boggess M.M., Watson J.F., Guest M., Duncanson K., Pezdirc K., Rollo M., Hutchesson M.J., Burrows T.L. (2014). Reproducibility and comparative validity of a food frequency questionnaire for Australian adults. Clin. Nutr..

[78] Collins C.E., Burrows T.L., Rollo M., Boggess M.M., Watson J.F., Duncanson K., Guest M., Pezdirc K., Hutchesson M.J. (2015). The comparative validity and reproducibility of a diet quality index for adults: The Australian Recommended Food Score. Nutrients.

[79] Leghi G.E., Middleton P.F., Netting M.J., Wlodek M.E., Geddes D.T., Muhlhausler B.S. (2020). A Systematic Review of Collection and Analysis of Human Milk for Macronutrient Composition. J. Nutr..

[80] Simmons D., Hague W.M., Teede H.J., Cheung N.W., Hibbert E.J., Nolan C.J., Peek M.J., Girosi F., Cowell C.T., Wong V.W.-M. (2018). Hyperglycaemia in early pregnancy: The Treatment of Booking Gestational diabetes Mellitus (TOBOGM) study. A randomised controlled trial. Med. J. Aust..

[81] Ellis K.J., Yao M., Shypailo R.J., Urlando A., Wong W.W., Heird W.C. (2007). Body-composition assessment in infancy: Air-displacement plethysmography compared with a reference 4-compartment model. Am. J. Clin. Nutr..

[82] Forsum E., Olhager E., Törnqvist C. (2016). An Evaluation of the Pea Pod System for Assessing Body Composition of Moderately Premature Infants. Nutrients.

[83] Villar J., Altman D.G., Purwar M., Noble J.A., Knight H.E., Ruyan P., Cheikh Ismail L., Barros F.C., Lambert A., Papageorghiou A.T. (2013). The objectives, design and implementation of the INTERGROWTH-21st Project. BJOG.

[84] De Onis M., Blossner M., World Health Organization WHO Global Database on Child Growth and Malnutrition. https://apps.who.int/iris/bitstream/handle/10665/63750/WHO_NUT_97.4.pdf.

[85] Thompson A.L., Mendez M.A., Borja J.B., Adair L.S., Zimmer C.R., Bentley M.E. (2009). Development and validation of the Infant Feeding Style Questionnaire. Appetite.

[86] Buysse D.J., Reynolds C.F., Monk T.H., Berman S.R., Kupfer D.J. (1989). The Pittsburgh sleep quality index: A new instrument for psychiatric practice and research. Psychiatry Res..

[87] Brouwers C.F.S. (2016). Obesity in the Pacific Island Countries a Literature Study on the Main Factors Contributing to the Extreme Prevalence of Obesity in Pacific Island Countries and the Nutrition Transition Model.

[88] Rueda-Clausen C.F., Poddar M., Lear S.A., Poirier P., Sharma A.M. (2020). Canadian Adult Obesity Clinical Practice Guidelines: Assessment of People Living with Obesity. Canadian Adult Obesity Clinical Practice Guidelines.

[89] Caleyachetty R., Barber T.M., Mohammed N.I., Cappuccio F.P., Hardy R., Mathur R., Banerjee A., Gill P. (2021). Ethnicity-specific BMI cutoffs for obesity based on type 2 diabetes risk in England: A population-based cohort study. Lancet Diabetes Endocrinol..

[90] Rasmussen K.M., Yaktine A.L. (2009). Weight Gain during Pregnancy: Reexamining the Guidelines.

[91] Beran T.N., Violato C. (2010). Structural equation modeling in medical research: A primer. BMC Res. Notes.

[92] Simpson S.J., Raubenheimer D. (2011). The nature of nutrition: A unifying framework. Aust. J. Zool..

[93] Team R.C. (2022). R: A Language and Environment for Statistical Computing.

[94] Whiteman V.E., Salemi J.L., Mejia De Grubb M.C., Ashley Cain M., Mogos M.F., Zoorob R.J., Salihu H.M. (2015). Additive effects of pre-pregnancy body mass index and gestational diabetes on health outcomes and costs. Obesity.

[95] Epstein L.H., Myers M.D., Raynor H.A., Saelens B.E. (1998). Treatment of Pediatric Obesity. Pediatrics.

[96] Avenell A., Brown T.J., McGee M.A., Campbell M.K., Grant A.M., Broom J., Jung R.T., Smith W.C.S. (2004). What interventions should we add to weight reducing diets in adults with obesity? A systematic review of randomized controlled trials of adding drug therapy, exercise, behaviour therapy or combinations of these interventions. J. Hum. Nutr. Diet..

[97] Watson L.C., Zimmerman S., Cohen L.W., Dominik R. (2009). Practical Depression Screening in Residential Care/Assisted Living: Five Methods Compared with Gold Standard Diagnoses. Am. J. Geriatr. Psychiatry.

[98] Perales M., Refoyo I., Coteron J., Bacchi M., Barakat R. (2015). Exercise during Pregnancy Attenuates Prenatal Depression. Eval. Health Prof..

[99] Harrod C.S., Chasan-Taber L., Reynolds R.M., Fingerlin T.E., Glueck D.H., Brinton J.T., Dabelea D. (2014). Physical activity in pregnancy and neonatal body composition: The Healthy Start study. Obstet. Gynecol..

